# Reversible Humidity-Driven Transformation of a Bimetallic {EuCo} Molecular Material: Structural, Sorption, and Photoluminescence Studies

**DOI:** 10.3390/molecules26041102

**Published:** 2021-02-19

**Authors:** Jakub J. Zakrzewski, Michal Heczko, Robert Jankowski, Szymon Chorazy

**Affiliations:** Faculty of Chemistry, Jagiellonian University, Gronostajowa 2, 30-387 Kraków, Poland; jakub.j.zakrzewski@doctoral.uj.edu.pl (J.J.Z.); michal.heczko@uj.edu.pl (M.H.); robert14.jankowski@doctoral.uj.edu.pl (R.J.)

**Keywords:** lanthanides, transition metals, cyanido ligand, luminescence, molecular switches

## Abstract

Functional molecule-based solids built of metal complexes can reveal a great impact of external stimuli upon their optical, magnetic, electric, and mechanical properties. We report a novel molecular material, {[Eu^III^(H_2_O)_3_(pyrone)_4_][Co^III^(CN)_6_]}·*n*H_2_O (**1**, *n* = 2; **2**, *n* = 1), which was obtained by the self-assembly of Eu^3+^ and [Co(CN)_6_]^3−^ ions in the presence of a small 2-pyrrolidinone (pyrone) ligand in an aqueous medium. The as-synthesized material, **1**, consists of dinuclear cyanido-bridged {EuCo} molecules accompanied by two H-bonded water molecules. By lowering the relative humidity (RH) below 30% at room temperature, **1** undergoes a single-crystal-to-single-crystal transformation related to the partial removal of crystallization water molecules which results in the new crystalline phase, **2**. Both **1** and **2** solvates exhibit pronounced Eu^III^-centered visible photoluminescence. However, they differ in the energy splitting of the main emission band of a ^5^D_0_ → ^7^F_2_ origin, and the emission lifetime, which is longer in the partially dehydrated **2**. As the **1** ↔ **2** structural transformation can be repeatedly reversed by changing the RH value, the reported material shows a room-temperature switching of detailed luminescent features including the ratio between emission components and the emission lifetime values.

## 1. Introduction

The search for novel dynamic functional solids is one of the trending topics in the field of materials science [1,2]. This highly desired group of materials exhibits various optical, magnetic, and/or electric properties which can be modulated by applying diverse external stimuli such as temperature [3], pressure [4], light irradiation [5], chemical species [6], and electric or magnetic field [7,8]. Whereas a linear response is usually the most desired from the viewpoint of sensing applications, the switchable materials exhibiting the abrupt structural transformations under the defined external conditions may be utilized in the construction of memory devices [9].

Within current trends in the area of dynamic functional solids, considerable attention is given to metal–organic frameworks (MOFs) or, more generally, to coordination polymers (CPs) [10,11]. They serve as an efficient platform for rational design of desired physical properties that takes advantage of a proper selection of metal centers and organic ligands at the synthetic stage [12]. This research pathway allowed chemists to obtain a large group of outstanding MOF/CP materials, as exemplified by photo-switchable magnetic CPs [13], MOF-based luminescent thermometers [14], and proton conductors sensitive to guest molecules [15]. It was also found accessible to obtain discrete, strictly molecular systems showing the analogous physical features as CPs of higher dimensionality [16,17]. In this regard, spin-crossover (SCO) and electron transfer (ET) molecular assemblies were fruitfully investigated from the viewpoint of temperature- and light-induced modulation of magnetic and absorption properties [18,19]. Moreover, functional molecular systems have been also recently recognized as promising materials extremely sensitive to many types of external stimuli. They include the presence of solvent vapors and variable external pressure which are the stimuli typically used for the tuning of physical properties of MOF materials and inorganic solids, respectively [20,21].

Aiming at the rational design of novel functional materials, we and other groups take advantage of cyanido metal complexes, which are accessible for most of the transition metals [22,23]. Some of them intrinsically exhibit attractive physical phenomena, such as reversible photo-reactivity [24] or vis-to-NIR photoluminescence [25,26], present in the solid-state and/or in the solution. Metal cyanido complexes, which are in the anionic form, can be readily combined with additional d/f-block metal cations, forming bimetallic coordination compounds, and such a conjunction of molecular building blocks was found to be a great pathway toward multifunctional molecule-based materials combining diverse optical, magnetic, and magneto-optical properties [27,28]. In particular, heterometallic cyanido-bridged assemblies are often sensitive to physical and chemical stimuli as illustrated by pressure- and electric-field-responsive CPs [29,30] or magnetic molecular cluster systems responding to solvent vapors, pressure, and temperature [31]. The reversible exchange of guest molecules was found to show a great impact on magnetic phenomena in cyanido-bridged bimetallic assemblies, including long-range spin ordering effects [32], single-molecule magnet (SMM) behavior [33], or even enabling a photo-switching of magnetic properties [34].

The appropriate selection of the specific cyanido metal complex can either introduce a new physical property, e.g., redox activity or photosensitivity [24,35], or improve the existing properties of a final material which is exceptionally realized for the combination of polycyanidometallates with lanthanide ions into d-f coordination frameworks and molecules [25,36]. Such conjunction may not only enable the observation of lanthanide-centered UV–vis photoluminescence but can also result in the effective sensitization of the 4f-centered emission throughout the radiationless energy transfer process involving d-d/charge-transfer electronic states of cyanido-based molecular moieties [37,38,39,40,41,42]. In this context, in the last few years, we focused our attention on the design of novel emissive d-f cyanido-bridged coordination systems aiming at multifunctional luminescent molecule-based materials linking photoluminescent features with single-molecule magnetism, proton conductivity, and solvent sorption properties [43,44,45]. Thus, we contributed to the more general research idea of multimodal functionalization of luminescent lanthanide molecular systems including their exploration as magnetic luminescent thermometers [46], luminescent molecular ferroelectrics [47], or luminescent ionic conductors [48]. Very recently, we reported a molecular material consisting of dinuclear {Dy^III^–[Co^III^(CN)_6_]^3−^} molecules exhibiting humidity-driven structural transformation enabling the efficient switching of the SMM behavior [49]. It exhibits also yellow Dy^III^-centered emission, slightly amended by the solvent content, but the luminescence was only limited to very low temperatures due to the generally weak luminescent character of this 4f metal ion. To overcome this limitation, we decided to focus on analogous cyanido-bridged molecules exploring red-emissive Eu^3+^ ions which are known to exhibit much stronger photoluminescence related both to the highly emissive ^5^D_0_ state as well as to the well-recognized emission sensitization by using the electronic properties of attached organic ligands [50,51]. Therefore, we report the crystal structures, optical and water sorption properties of a novel molecular material {[Eu^III^(H_2_O)_3_(pyrone)_4_][Co^III^(CN)_6_]}·*n*H_2_O (**1**, *n* = 2; **2**, *n* = 1; pyrone = 2-pyrrolidinone) based on dinuclear cyanido-bridged {EuCo} molecules and hydrogen-bonded water molecules. This material exhibits two variously solvated crystalline phases, **1** and **2**, reversibly interconvertible by the change of relative humidity which results in a subtle modulation of room-temperature visible light photoluminescence characteristics. These effects were investigated using X-ray diffraction methods, dynamic vapor sorption studies, and thorough analysis of emission properties.

## 2. Results and Discussion

### 2.1. Basic Characterization and Structural Studies

The combination of europium(III) chloride and potassium hexacyanidocobaltate(III) in the water/2-pyrrolidinone mixture afforded colorless crystals of **1**, which were found to exhibit bright red luminescence under UV light irradiation indicating the presence of Eu^III^ complexes (see Experimental section for details). Due to the high sensitivity of the obtained material to the variation of relative humidity, which was also observed in the previously reported, analogous {DyCo} molecular system [49], the preliminary characterization of **1** was performed with the appropriate precautions to control the solvent content of the material. The IR spectra and the CHN elemental analysis confirmed the presence of two expected crystalline phases depending on the humidity conditions (Figure S1, Table S1, and Experimental section). The results of thermogravimetric analysis (TGA) allowed us to estimate the water content and define the conditions for the dehydration of the single-crystal of **1** within the X-ray diffraction (SC-XRD) experiment (Figure S2 and the related comment). Initially, the crystal structure determination was performed for the selected single crystal of **1** taken directly from the mother solution. Then, the temperature was elevated to 320(2) K, and the structural transformation to **2** was performed in situ, which was followed by the SC-XRD experiment for the dehydrated crystal of **2** (see Experimental section for details). Both phases were found to build the periodic structure within the $P\bar{1}$ space group belonging to a triclinic crystal system (Table 1).

**Figure 1:**
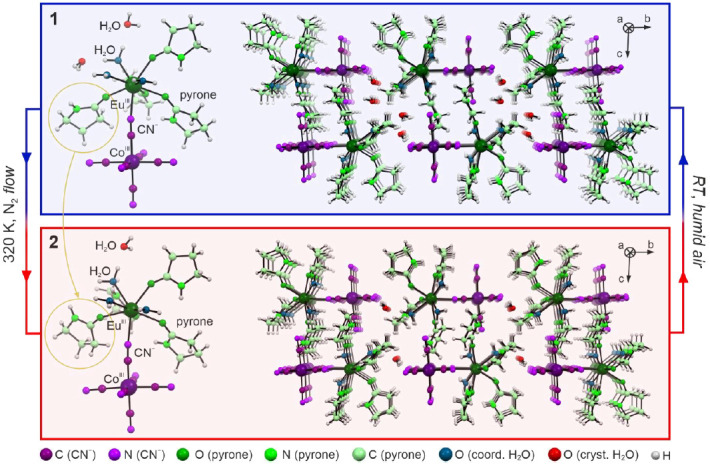
Representative views of the crystal structures of **1** and **2**. The graphic presents molecular building units of both structures (**left side**), and the crystal packing along *a* crystallographic axis (**right side**). The rotation of the pyrone ligand is highlighted by yellow circles. The experimental conditions of the observed transformation for a single crystal were placed in the figure. The Co- and Eu-atoms are labeled in the figure while the color code for all other atoms is shown at the bottom.

**1** and **2** are composed of dinuclear cyanido-bridged molecules characterized by a composition of [Eu(H_2_O)_3_(pyrone)_4_(μ-NC)Co(CN)_5_] (pyrone = 2-pyrrolidinone). They are accompanied by two additional water molecules of crystallization per the {EuCo} formula unit for **1**, while only one such molecule is present in **2** (Figure 1, Figure S3 and Tables S2–S4). The first coordination sphere of Eu^III^ complex consists of three O-atoms of coordinated water molecules, four O-atoms from the carbonyl groups of organic ligands, and one cyanido N-atom of the Eu–NC–Co molecular linkages. Octahedral Co^III^ center, placed on the second side of the molecular bridge, coordinates six C-bonded cyanido ligands. Within the molecular part of the structure, **1** and **2** differ mainly in detailed structural features. To precisely determine the coordination geometry of Eu^III^ complexes in both phases, Continuous shape measure (CSM) analysis was performed (Table S4). In **1** and **2**, eight-coordinated Eu^III^ complexes are present, and they reveal a mixed geometry between a dodecahedron (TDD-8) and a biaugmented trigonal prismatic (BTPR-8). For **1**, the CSM parameter related to the TDD-8 geometry is significantly closer to the ideal shape than the one for the BTPR-8 geometry. After the structural transformation to **2**, both CSM parameters are almost equal with a small advantage toward a dodecahedral geometry. This is in contrast to the report concerning analogous {DyCo} molecules [49], where the structural transformation resulted in more pronounced conversion within the first coordination sphere, from the distinct TDD-8 to the closely ideal BTPR-8. This effect is presumably caused by a bigger ionic radius of Eu^3+^ ions, which results in their smaller response to the solvent-driven supramolecular rearrangement of the crystal lattice.

As mentioned above, the structural transformation between **1** and **2** is related to the removal of one crystallization water molecule per {EuCo} formula unit, which mainly affects supramolecular interactions realized by hydrogen bonds (Figure S4 and Tables S5 and S6). In **1**, one of the coordinated aqua ligands forms H-bonds with two present crystallization water molecules. They further interact with terminal cyanido ligands and with each other. After the partial removal of the solvent, phase **2** includes only one water molecule being the donor of two H-bonds toward cyanido ligands. The final structure of **2** lacks voids as, in its crystal structure, the same aqua ligand previously bonding two H_2_O molecules forms close contact with one of the terminal CN^−^ ligands. This leads to the significant rearrangement of mutual positions of the neighboring {EuCo} molecules. As a result, the initial closest distance between Ln centers in **1** of 9.0135(6) Å decreases in **2** down to 8.112(1) Å.

Apart from this, only a slight increase of Eu–Co distance (ca. 0.6%) is observed with the partial dehydration which results from a small variation in the CN–Eu binding angle. There is also a shift in the ligand orientation which is marked in Figure 1.

This is caused by the breakage of the H-bond related to the amine H-atom, and the subsequent repositioning of the ligand in the vacant space appearing in the crystal structure of **2**. This structural modification finds also a reasonable reflection in the IR spectra (Figure S1) showing the noticeable changes in the region assignable to the stretching vibrations of N–H groups of pyrone ligands while the energies of cyanide stretching vibrations only subtly vary upon transformation between **1** and **2** (Table S1 and Experimental section).

The described differences in the structures of **1** and **2** result in a slight decrease of the unit cell volume occurring upon the structural transformation. The most pronounced changes are observed within the unit cell angles (Table 1). The variation of the unit cell allows us to easily distinguish both phases using the powder X-ray diffraction (P-XRD) experiment. Using this method, the phase purity and the validity of the applied structural models were confirmed (Figure S5). Similarly to the {DyCo} analog, the {EuCo} molecular system can be repeatedly interconverted between **1** and **2** phases without a significant loss of crystallinity. However, the subsequent hydration/dehydration cycles noticeably reduce the quality of the investigated single-crystal due to increased structural disorder and crystal mosaicity resulting from the partial removal of the solvent from the structure [43].

### 2.2. Sorption Properties

**Figure 2:**
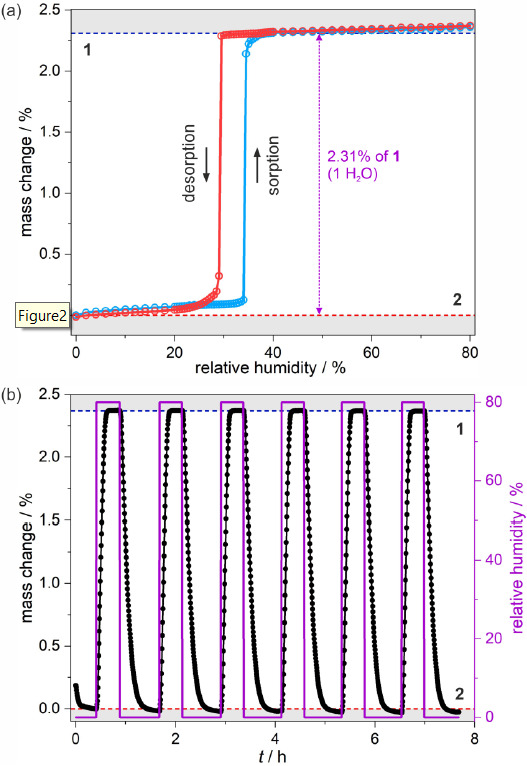
Room-teFigmperature humidity-driven switching between 1 and 2 illustrated by dynamic vapor sorption experiments: water sorption (blue line) and desorption (red line) isotherms measured at 298 K with the indicated mass change (violet arrow) (**a**), and the related hydration/dehydration cycles (**b**), both performed in the 0–80% range of the relative humidity. In (**b**), black and violet curves represent changes in the sample mass and the humidity level, respectively.

Thermogravimetric analysis indicates that the sample of **1** readily loses the part of crystallization water molecules when placed into the flow of dry nitrogen. This behavior, together with the possibility to perform the reverse transformation from **2** to **1** by exposure to the humid atmosphere, e.g., in a closed vial partially filled with water, implies that the obtained {EuCo} molecular system should exhibit water sorption properties. To perform the reliable analysis of a sorptive character of **2**, the dynamic vapor sorption (DVS) method was applied (see Experimental section for details). The powder sample of **1** was inserted into the chamber of the apparatus and dehydrated in situ using a pure N_2_ flow at room temperature. The isothermal sorption and desorption curves at 298 K indicate the formation of both phases in the relative humidity (RH) range of 0–80% (Figure 2).

At 0 RH, **2** is a stable phase as no further dehydration, involving coordinated water molecules or even the second crystallization solvent molecule, occurs. The small elevation of RH slightly affects the sample mass due to the physical adsorption, which persists at up to 34% RH. Then, even a small change in humidity of 0.5% results in abrupt water uptake. At this stage, the majority of the sample transforms into phase **1**, but the finite size of microcrystals hampers full conversion, which requires elevation of RH up to ca. 38%. During the conversion upon the RH change from 0 to ca. 38%, the sample increases its weight by 2.31% which corresponds to ca. 1 water molecule per the {EuCo} formula unit, but the weight increase within the abrupt step is slightly smaller. This means that upon initiation of the solvation process, the initially physically adsorbed water is transferred into the crystal lattice which is noticed as seemingly drying of the powder when the hydration of **2** into **1** is realized outside of the DVS apparatus. The increase of humidity above 40% RH leads to weaker physical adsorption, which continues up to 80% RH. Treating the sample with the higher humidity results in the accumulation of water vapor related to the dissolution of the microcrystals of **1** in pure H_2_O. The reverse curve of isothermal desorption resembles the initial curve.

But the humidity-driven structural transformation from **1** to **2** appears at a lower RH value. This results in the appearance of a humidity-oriented hysteresis loop. Upon decreasing the humidity, sorption and desorption curves follow the same trend in the 80–38% RH range but, then, the sample remains in phase **1** down to 29% where the water excess is removed in an almost one abrupt step. Symmetrically to the hydration process, the desorption curve indicates full structural conversion to **2** with a visible shift down to ca. 24%, which is followed by the recreation of an initial sorption step. Besides the sorption/desorption isothermal experiment, several cycles of hydration and dehydration of the sample in the 0–80% range were performed proving the perfect reversibility of the sorption-related processes (Figure 2b).

The results of the water sorption studies reveal that the structural transformation related to the content of crystallization water molecules can be induced solely by the RH change at room temperature. While a similar sorption-induced behavior was previously found for the Dy-based analog, here the {EuCo} molecular system reveals the hysteretic behavior in the much lower relative humidity range. In the former compound, the conversion from the low-humidity phase starts at 47% RH, and the abrupt desorption step occurs below 42%. This large shift of more than 10% RH in the hysteresis position between two different incorporated Ln^3+^ ions can be assigned to the modulation in metal ionic radius, which influences the critical structural features of the final material.

### 2.3. Photoluminescent Properties

**Figure 3:**
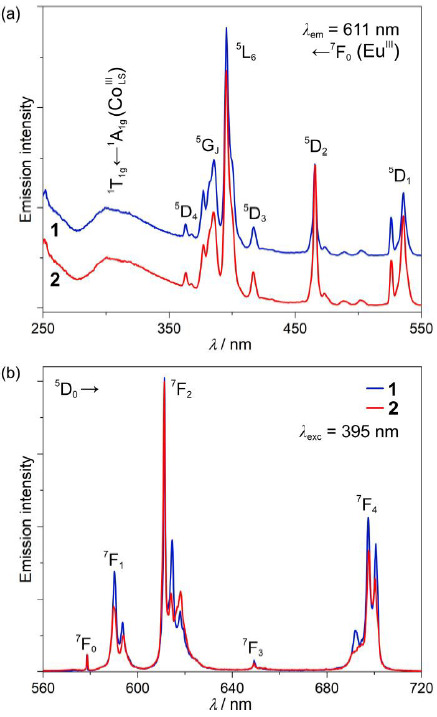
Room-temperature emission properties of **1** and **2**. Excitation spectra monitored at *λ*_em_ = 611 nm (**a**), and emission spectra measured under irradiation with *λ*_exc_ = 395 nm (**b**). The main electronic transitions are assigned in the figure.

Thanks to the presence of Eu^III^ complexes, under the UV-light irradiation, **1** and **2** reveal room-temperature red emission originating from f-f electronic transitions. Searching for the differences in the photoluminescence characteristics between these two phases, excitation and emission spectra were measured for carefully prepared powder samples of **1** and **2** (Figure 3 and Figure S6). The excitation spectra of **1** and **2** are dominated by a series of sharp peaks in the 350–550 nm range, which can be assigned to the direct excitation through the f-f electronic excited states of Eu^III^. The most intensive maximum is located at ca. 395 nm, and it corresponds to the ^5^L_6_ ← ^7^F_0_ electronic transition [52], but the existence of the broad band located in the 270–350 nm region should also be noted. The latter originates from a moderate energy transfer (ET) process occurring from d-d electronic states of Co^III^_LS_ centers of the cyanido-bridged [Co(CN)_6_]^3−^ units. The efficiency of this ET process is rather weak as it is overpassed by the Eu^III^-centered direct excitation pathways. This is due to the relatively long intermetallic Eu–Co distances, the 1:1 Co-to-Eu stoichiometry in the obtained phases and, thus, the attachment of only a single Co^III^ center for each Eu^III^ site in addition to the presumable occurrence of an energy back-transfer effect at room temperature [53,54]. It is difficult to distinguish **1** and **2** using the excitation spectra as the excited states of Ln^3+^ ions are barely affected by the coordination geometry, and the presented structural transformation seems to be irrelevant in terms of the observed Co-to-Eu energy transfer. Simultaneously, the emission spectra of both **1** and **2** contain only a series of characteristic for Eu^III^ complexes emission lines, which are assignable to the f-f ^5^D_0_ → ^7^F_J_ (*J* = 0, 1, 2, 3, 4) electronic transitions, but with a noticeable sub-structure within the most intense maxima located at ca. 580, 615, and 700 nm (Figure 3b). The splitting of Eu^III^-centered emission bands varies between **1** and **2**, but it is also slightly affected by the selection of the excitation pathway (Figure S6). Nevertheless, there is no broad emission pattern in the visible range that could be expected for red-phosphorescent [Co(CN)_6_]^3−^ units under the 300 nm excitation [54,55], confirming the occurrence of at least partial Co-to-Eu energy transfer process.

The most prominent differences between the spectra for **1** and **2** are visible in the most intense maxima related to the ^5^D_0_ → ^7^F_2_ electronic transition. Here, the intensity ratio between the maxima located at 614.7 and 618.2 nm changes from ca. 2.2 in **1** to ca. 1.0 in **2**. To check the origin of the selected maxima, the high-resolution emission spectra of **1** under the *λ*_exc_ = 395 nm excitation were measured at low temperatures (*T* = 30 K, Figure S7). They reveal that the pattern of the observed emission spectrum at room temperature is mainly determined by the *m*_J_ electronic sub-structure of the respective multiplets with a typical broadening of the respective emissive bands at higher temperatures but without any easily distinguishable hot transitions. This means that the difference between the two indicated emission components of **1** and **2** is mainly related to the changes in the transition probability between the respective *m*_J_ levels within the emissive and the ground multiplets. This can be assigned to the subtle modulation of the coordination geometry of Eu^III^ complexes in **1** and **2** which can dramatically influence the detailed state composition of *m*_J_ levels [45].

**Figure 4:**
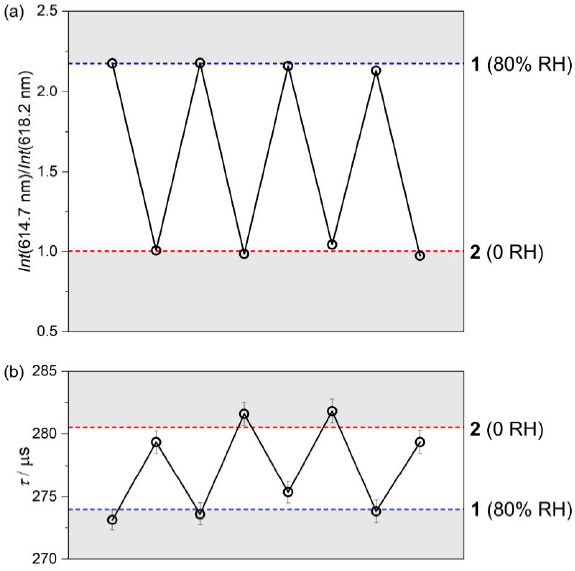
Repeatability of the emission signal in the subsequent dehydration/hydration cycles, performed at room temperature, presented using the intensity ratio between the emission maxima located at 614.7 and 618.2 nm (**a**) and using the values of the extracted emission lifetimes (**b**). The related emission decay profiles were presented in Figure S8 in the Supplementary Materials. Blue and red dashed lines indicate 0 and 80% relative humidity values, respectively.

To check the repeatability of the emission signal upon the reversible structural transformation between **1** and **2**, the emission spectra under the 395 nm light irradiation were measured in a few cycles of dehydration and hydration, and the characteristic intensity ratio between the 614.7 and 618.2 nm lines was found to be perfectly reproducible (Figure 4).

In this context, we also examined how the observed humidity-driven transformation affects the room-temperature emission lifetimes measured upon the optimal excitation wavelength of 395 nm, for the monitored maximum emission at 611 nm (Figure S8). The resulting average *τ* values w 274.0(7) μs and 280.5(2) μs for **1** and **2**, respectively, are intermediate among Eu^III^ complexes indicating rather moderate overall emission efficiency, and the presence of significant emission de-activation pathways, which can be ascribed to the large number of O–H, C–H, and N–H oscillators in close vicinity of emissive Eu^III^ centers [50,51,52]. The small but readily noticeable lengthening of emission lifetime after the partial dehydration can be assigned to the reduction of the amount of O–H oscillators within the crystal structure of **2**. Similarly to the emission itself, the emission lifetime was also found reproducible in the analogous cycles of dehydration and hydration. However, the smaller variation of emission lifetime makes this parameter less reliable as a sign of occurring humidity-driven structural transformation (Figure 4). Nevertheless, both optical parameters, the ratio between two distinct emission components and emission lifetime, efficiently illustrate the difference in optical properties of two studied crystalline phases and thus can be applied to distinguish **1** and **2** without the involvement of XRD techniques. The conclusion on the moderate emission efficiency both in **1** and **2** was also confirmed by the determination of the absolute quantum yield (QY). Under the 395 nm irradiation, related to the direct f-f excitation pathway, the QYs are 4.0(2)% and 7.1(3)% for **1** and **2**, respectively. They are rather small when compared with the most efficient Eu^III^-based luminophores [52]. The increase of the QY upon the partial dehydration of **1** into **2** stays also in agreement with the emission lifetime measurements and can be also assigned to the reduction of the amount of O–H oscillators in close vicinity of Eu^III^ centers in **2**. We also determined the emission QYs for the 300 nm irradiation related to the excitation band assignable to [Co^III^(CN)_6_]^3−^ ions (Figure 3a). The resulting values of 1.6(1)% and 0.8(3)% for **1** and **2**, respectively, are much smaller than the respective values obtained under the direct f-f excitation which agrees with the observed differences in the intensity of the related excitation bands (Figure 3a). Interestingly, it seems to be better in the hydrated phase **1** than in the partially dehydrated phase **2**. This can be correlated with the Eu1–Co1 distance of 5.4261(4) Å in **1** which is shorter than the value of 5.4583(9) Å in **2** (Table S1), thus facilitating a slightly more efficient metal-to-metal energy transfer.

## 3. Conclusions

By employing strongly photoluminescent Eu^3+^ ions together with hexacyanidocobaltate(III) metalloligands and small blocking 2-pyrrolidinone (pyrone) ligands, a novel dynamic material based on dinuclear {EuCo} molecules, showing humidity-level-variable room-temperature photoluminescence, was obtained. Two easily interconvertible crystalline phases differing in the amount of crystallization water molecules, **1** and **2**, were formed depending on the level of humidity in ambient conditions. They exhibit pronounced Eu^III^-based emission with distinguishable optical characteristics, including the ratio between two emission components and the values of emission lifetimes. As a result, the humidity-driven structural transformation between two solvates of the obtained material enables the switching of specific luminescent features. The emission switching was found to be reproducible in a series of subsequent cycles of dehydration and hydration which is related to the sorptive ability of the obtained molecule-based system, as illustrated by water vapor sorption studies correlated with X-ray diffraction structural analysis. Moreover, the detailed investigation of sorption properties reveals the phase bistability effect occurring in the 29–34% RH region which indicates that two distinguishable states of the molecular system can be stable under identical conditions e.g., 32% RH, which gives an unusual type of a memory effect. Taking into account the previous report on the analogous {DyCo} molecules [49], the described molecule-based material shows significant changes in the sorption properties depending on the size of the employed f-block metal ion. The introduction of a bigger lanthanide ion, Eu^3+^ over previously used Dy^3+^, promotes the stability of the as-synthetized phase with two water molecules per {LnCo} formula unit. It looks that the humidity level, for which the dehydration-related transformation occurs, can be rationally designed by playing with the ion size or the mixtures of 4f metal ions in the final material. Due to the lack of energy transfer from organic ligands and rather inefficient sensitization of the Eu-based emission by Co^III^_LS_ centers, the changes within the hexacyanidometallate subunits seems critical for the eventual observation of photoluminescence at room temperature for the analogs with different Ln^3+^ ions. However, such change would also affect the response of the material to the humidity level as terminal cyanido ligands are richly engaged in the formation of the hydrogen-bonding networks of both reported crystalline phases. This opens great prospects for the further tuning of water sorption properties, the broadening toward reversible uptake of other small molecules (such as methane, ammonia, or carbon dioxide), and the resulting switching of diverse luminescent features by using dinuclear {Ln(pyrone)–[M(CN)_6_]^3−^} molecule-based materials and the related lanthanide–hexacyanidometallate molecular systems.

## 4. Experimental Section

### 4.1. Materials

Europium(III) chloride hexahydrate, EuCl_3_·6H_2_O (CAS: 13759-92-7), 2-pyrrolidinone (pyrone, CAS: 616-45-5), and potassium hexacyanidocobaltate(III), K_3_[Co(CN)_6_] (CAS: 13963-58-1) were purchased from Sigma-Aldrich and used without further purification.

### 4.2. Synthesis and Basic Characterization of **1**

The 0.96 mmol (351.5 mg) portion of EuCl_3_·6H_2_O was dissolved in the solution containing 16.5 mmol (1.403 g) of liquid 2-pyrrolidone and 2 mL of distilled water. After that, the 1.00 mmol (332.3 mg) portion of K_3_[Co(CN)_6_] was dissolved in the 2 mL of distilled water. Both solutions were combined, vigorously stirred for ca. 30 s, and filtered. The resulting clear filtrate was left for crystallization. After a few minutes, the colorless crystals of **1** start to appear at the bottom of the vial, but the mixture was left undisturbed in the darkness for ca. 1 day. The obtained crystalline phase is stable in the mother solution, and it is air-stable if the relative humidity exceeds 37% at 298 K, as demonstrated by the water sorption studies (Figure 2). The yield of the synthesis (ca. 40–50%) varies between the samples as compound **1** easily dissolves in distilled water which needs to be used for washing the crystals after their removal from the mother solution. The formula of **1**, {[Eu(H_2_O)_3_(pyrone)_4_][Co(CN)_6_]}·2H_2_O (*M*_W_ = 797.50 g·mol^−1^), was determined using the single-crystal X-ray diffraction analysis and confirmed by the results of CHN elemental analysis. Anal. calcd: C, 33.1%; H, 4.8%, N, 17.6%. Found: C, 33.2%, H, 4.7%; N, 17.7%. IR spectrum (Figure S1; cm^−1^): 2102, 2130, 2142, 2150, 2160—cyanide stretching vibrations; 1653 and 1685—stretching vibrations of the carbonyl group (pyrone ligands); and an intense broad band above 3000 is related to the presence of H-bonded water molecules with a small maximum at 3374 caused by the stretching vibrations of N–H groups (pyrone ligands) [56]. The energies of the part of cyanide stretching vibrations significantly exceeds the value of 2129 cm^−1^ found for the K_3_[Co(CN)_6_] precursor confirming the presence of bridging cyanido mode (Table S1) [57]. They are also very similar to those detected in other dinuclear lanthanide(III)–hexacyanidometallate(III) molecules (Table S1) [45,57].

### 4.3. Preparation and Basic Characterization of **2**

To prepare the sample of **2**, the crystals of **1** have to be removed from the mother solution by suction filtration, washed with a small amount of water and ethanol, and dried under inert gas (Ar or N_2_). Phase **2** is generally stable when stored at the humidity level below 25% at 298 K and when protected by Apiezon^®^ N grease or paraffin oil. The formula of **2**, {[Eu(H_2_O)_3_(pyrone)_4_][Co(CN)_6_]}·H_2_O (*M*_W_ = 779.48 g·mol^−1^), was determined using the single-crystal X-ray diffraction analysis, and further confirmed by the results of CHN elemental analysis performed under the appropriate humidity conditions. Anal. calcd: C, 33.9%; H, 4.7%, N, 18.0%. Found: C, 34.0%, H, 4.5%; N, 18.1%. IR spectrum (Figure S1; cm^−1^): 2105, 2133, 2137, 2151, 2162—cyanide stretching vibrations; 1647 and 1687—stretching vibrations of the carbonyl group (pyrone ligands); and an intense broad band above 3000 is related to the presence of H-bonded water molecules with a small maximum at 3403 caused by the stretching vibrations of N–H groups (pyrone ligands) [56]. The cyanide stretching vibrations are only slightly modified when compared with those found for **1** (Table S1), thus a more significant impact of the transformation from **1** to **2** is observed in the higher-energy vibrations of N–H groups, which stays in good agreement with the related humidity-driven rearrangement in the supramolecular framework involving pyrone ligands (see Structural Studies in Section 2.1 for details).

### 4.4. Crystallographic Studies

Single-crystal X-ray diffraction (SC-XRD) analyses of **1** and **2** were performed using a Bruker D8 Quest Eco Photon50 CMOS (Billerica, MA, USA) equipped with graphite monochromated MoKα radiation. The single crystal of **1** was taken directly from the mother solution, dispersed in Apiezon^®^ N grease, mounted on a Micro Mounts^TM^ holder, and measured at the low temperature of 100(2) K. After that, the temperature was increased to 320(2) K and the crystal was kept for ca. 3 h in the N_2_ flow to fully transform the compound into phase **2**. The completeness of the structural transformation and further stability of **2** was checked by the several repetitions of the unit cell measurement at 320(2) K. The dehydration of **1** into **2** was finished when three consecutive unit cell measurements gave the unchanged results with identical indexing accuracy. Then, the in situ formed crystal of **2** was cooled down to 100(2) K and the SC-XRD experiment was performed. Crystal structures of both **1** and **2** were solved by an intrinsic phasing method using SHELXT-2014/5, and refined using a full-matrix least-squares technique of SHELXL-2018/3 [58]. The refinement procedure was conducted using WinGX (version 2014.1, Glasgow, UK) integrated software [59]. All non-hydrogen atoms were refined anisotropically. The hydrogen atoms of pyrone ligands were calculated on the idealized positions and refined using the riding model, while those of coordinated and crystallization water molecules were found from the electron density map and refined isotopically with DFIX and SADI restraints. The CCDC reference numbers for **1** and **2** are 2058372 and 2058373, respectively. CCDC contains the supplementary crystallographic data for this paper. These data can be obtained free of charge via http://www.ccdc.cam.ac.uk/conts/retrieving.html (or from the CCDC, 12 Union Road, Cambridge CB2 1EZ, UK; Fax: +44-1223-336033; E-mail: deposit@ccdc.cam.ac.uk). Structural figures were prepared using the Mercury 4.2.0 software (Cambridge, UK). Continuous shape measure analysis for the coordination sphere of eight-coordinated Eu^III^ complexes was performed using SHAPE software ver. 2.1. (Barcelona, Spain) [60]. The powder X-ray diffraction patterns (P-XRD) patterns were measured using a Bruker D8 Advance Eco diffractometer equipped with a CuKα radiation source and a capillary spinning add-on. Before the P-XRD measurement, the powder sample of **1** was stabilized for 2 h at the humidity level of 80% (N_2_ flow) in the PCV chamber using a humidity generator (HG-100 dew point generator; L&C Science and Technology, Hialeah, FL, USA) and loaded into a glass capillary. By contrast, the sample of **2** was loaded inside a glove box system after a few cycles of treatment with vacuum and Ar flow and then used for the P-XRD studies.

### 4.5. Physical Techniques

Preparation of the samples for detailed physical characterization was performed under very strict humidity conditions to generate the pure crystalline phases of **1** (stable for the relative humidity above 37%) or **2** (stable for the humidity level below 25%). It was particularly important as the typical relatively humidity of the laboratory oscillates around 30% (±10%), thus both crystalline phases could be observed. For spectroscopic studies (IR, room-temperature photoluminescence), the sample of **1** was prepared by stabilization at the 80% relative humidity while the sample of **2** was prepared by dehydration in the glove box chamber. Both methods were identical as described above for the P-XRD experiments. IR spectra were measured on the selected single crystals of **1** and **2** dispersed in the Apiezon^®^ N grease. A Nicolet iN10 MX Fourier transforms infrared microscope was used. CHN elemental analyses were performed on an Elementar Vario Micro Cube analyzer. Thermogravimetric (TG) analyses of **1** and **2** were carried out under a nitrogen atmosphere using TG209 F1 Libra thermogravimetric analyzer (Netzsch, Selb, Germany). The sample of **1** was measured using an Al_2_O_3_ pan with a lid while for **2** the standard Al pan was used (see Comment to Figure S2 for more details). Luminescent characteristics, including room-temperature solid-state emission and excitation spectra, were investigated using an FS5 spectrofluorometer (Edinburgh Instruments, Livingstone, UK) equipped with xenon (150 W) arc lamp as an excitation source and an R928P Hamamatsu photomultiplier as a detector. Emission lifetime measurements were conducted on the same apparatus employing an FS5 multichannel scaling module with a Xe flash lamp. For each measurement, including the repeatability of the emission signal upon the humidity changes, the samples were loaded after the same treatment as in the case of P-XRD studies. The temperature-variable emission spectra were collected on the same spectrofluorometer using a CS204SI-FMX-1SS cooling power optical helium cryostat (Advanced Research Systems, Inc., Macungie, PA, USA) equipped with a DE-204SI closed-cycle cryo-cooler (cold head), water-cooled He compressor (ARS-4HW model), and a cryogenic temperature controller (model 335, Lake Shore Cryotronics, Westerville, OH, USA). In this type of measurement, the powder sample of **1** was inserted in the transparent foil bag under the 80% relative humidity level and placed between two quartz plates. The background correction was performed within the Fluoracle software (Edinburgh Instruments, Livingstone, UK). Absolute emission quantum yields were determined using a direct excitation method with an integrating sphere module [61]. The water absorption isotherm was measured using the dynamic vapor sorption method using an SMS DVS Resolution apparatus in the humidity range of 0–80% (using the N_2_ flow) at 298 K. For this experiment, a freshly prepared sample of **1** was inserted into the apparatus and dehydrated under the dry nitrogen flow to obtain phase **2**. The full isotherm cycle consisted of sorption followed by desorption. The cycles of rehydration/dehydration were also performed at 298 K for the sample held at 0% or 80% RH until a stable mass was achieved. In all these experiments, a d*m*/d*t* value of 0.002% min^−1^ was chosen as the requirement for sample mass stability.

## Figures and Tables

**Table 1:** Crystal data and structure refinement for **1** and **2**.

Compound	1	2
formula	Eu_1_Co_1_C_22_H_38_N_10_O_9_	Eu_1_Co_1_C_22_H_36_N_10_O_8_
formula weight/g·mol^−1^	797.51	779.5
*T*/K	100(2)	
*λ*/Å	0.71073 (Mo Kα)	
crystal system	triclinic	
space group	$P\bar{1}$	
*a*/Å	9.0135(5)	9.032(1)
*b*/Å	12.0910(7)	12.089(2)
*c*/Å	14.3010(8)	14.023(2)
*α*/°	90.199(2)	95.815(4)
*β*/°	92.429(2)	90.186(4)
*γ*/°	90.912(2)	90.138(4)
*V*/Å^3^	1556.9 (2)	1523.2(4)
*Z*	2	
*ρ*_calc_/g·cm^−3^	1.701	1.700
*μ*/cm^−1^	2.592	2.645
*F*(000)	804	784
crystal type	colorless plate	
crystal size/mm × mm × mm	0.35 × 0.23 × 0.05	
*θ* range/°	2.725–25.026	2.345–25.027
limiting indices	−10 < *h* < 10−14 < *k* < 14−15 < *l* < 17	−10 < *h* < 10−14 < *k* < 14−16 < *l* < 16
collected reflections	15,970	16,135
unique reflections	5485	5354
*R*_int_	0.0185	0.0346
completeness/%	99.7	99.5
data/restraints/parameters	5485/15/428	5354/13/411
*GOF* on *F*^2^	1.097	1.153
*R*_1_ [*I* ≥ 2*σ*(*I*)]	0.0179	0.0319
w*R*_2_ (all data)	0.0444	0.0891
largest diff. peak/hole/e∙Å^−3^	0.842/−0.408	1.896/−1.306

## Data Availability

The data presented in this study are available on request from the corresponding author. The data are not publicly available as all essential results related to this work are already included in the manuscript and Supplementary Materials.

## Supplementary Materials

Figure S1, Table S1: IR spectra, Figure S2: TG curves, Table S2: selected structural parameters, Tables S3 and S4: CSM analysis, Figure S3: asymmetric units, Table S5–S6: hydrogen bond parameters, Figure S4: views of the H-bonds, Table S5: PXRD patterns, Table S6: additional RT emission spectra, Table S7: low-temperature emission spectrum of **1**, Figure S8: emission decay profiles.

## Sample Availability

Samples of the compounds **1** and **2** are available from the authors.
