# Supplementary material for: Reversible Humidity-Driven Transformation of a Bimetallic {EuCo} Molecular Material: Structural, Sorption, and Photoluminescence Studies
Source: Molecules. 2021 Feb 19;26(4):1102. doi: 10.3390/molecules26041102 (PMC7923019; doi:10.3390/molecules26041102)
Supplement: Supplementary file 1 [file molecules-26-01102-s001.zip › revised supporting information.pdf]

Supporting Information

Reversible Humidity-Driven Transformation of a Bimetallic {EuCo} Molecular Material: Structural, Sorption, and Photoluminescence Studies

Jakub J. Zakrzewski, Michal Heczko, Robert Jankowski, and Szymon Chorazy*

Faculty of Chemistry, Jagiellonian University, Gronostajowa 2, 30-387 Kraków, Poland.

*Corresponding author: chorazy@chemia.uj.edu.pl

IR spectra of **1** and **2**. (Figure S1) S2

Comparison of cyanide stretching vibrations in **1** and **2** with $K_3[Co(CN)_6]$ and the reported Ln–$[M(CN)_6]^{3-}$ (M = Co, Rh, Ir) dinuclear molecules. (Table S1) S2

Thermogravimetric curves of **1** and **2**. (Figure S2) S3

Comment to Figure S2. S4

Selected structural parameters of Eu^{III} complexes in **1** and **2**. (Table S2) S5

Selected structural parameters of Co^{III} complexes in **1** and **2**. (Table S3) S6

Results of CSM analysis for Eu^{III} complexes in the crystal structures of **1** and **2**. (Table S4) S7

Comparison between the asymmetric units of **1** and **2** with atoms labelling schemes. (Figure S3) S7

Detailed parameters of the hydrogen bonds in the crystal structure of **1**. (Table S5) S8

Detailed parameters of the hydrogen bonds in the crystal structure of **2**. (Table S6) S9

Detailed views of the hydrogen bonds within supramolecular networks of **1** and **2**. (Figure S4) S10

Powder X-ray diffraction patterns for **1** and **2**. (Figure S5) S11

Emission spectra of **1** and **2** gathered at room temperature upon 300 nm irradiation. (Figure S6) S12

Emission spectrum of **1** gathered at T = 30 K upon 395 nm irradiation. (Figure S7) S12

Emission decay profiles of **1** and **2** in subsequent cycles of hydration/dehydration. (Figure S8) S13

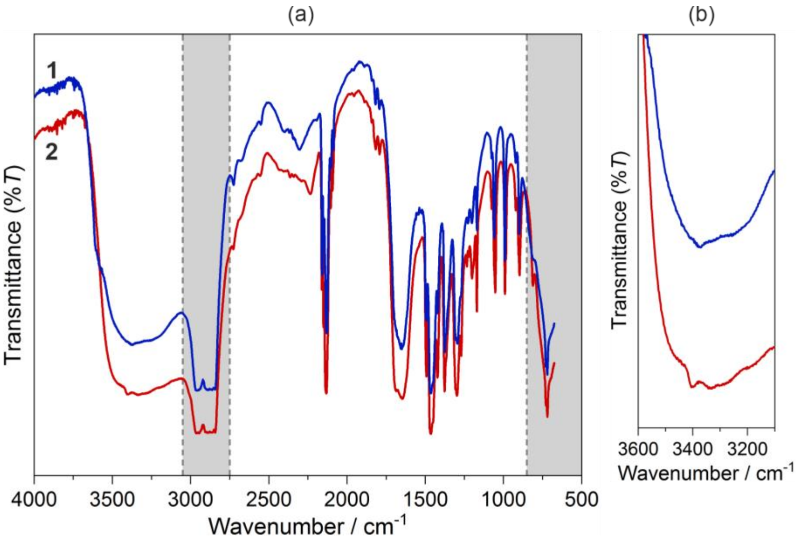

Figure S1. IR spectra of **1** and **2** measured in the 4000–700 cm^{-1} range (*a*) with the enlargement of the 3600–3100 cm^{-1} region related to the vibrations of H-bonded water molecules (broad component) and the stretching vibrations of N-H groups of pyrone ligands (small peaks on the broad bands) (*b*). The indicated grey area shows the range where the strong absorption from the protective medium (Apiezon® N grease) is observed.

Table S1. Comparison of cyanide stretching vibrations in **1** and **2** with $K_3[Co(CN)_6]$ and the reported Ln–$[M(CN)_6]^{3-}$ (M = Co, Rh, Ir) dinuclear molecules.

**Compound**	**Cyanide stretching vibrations / cm^{-1}**	**Reference**
**1**	2102, 2130, 2142, 2150, 2160	this work
**2**	2105, 2133, 2137, 2151, 2162	this work
$K_3[Co^{III}(CN)_6]$	2129	[S1]
$\{[Dy^{III}(H_2O)_3(pyrone)_4][Co^{III}(CN)_6]\}\cdot 2H_2O$	2123, 2130, 2142, 2150, 2162	[S2]
$\{[Dy^{III}(H_2O)_3(pyrone)_4][Co^{III}(CN)_6]\}\cdot H_2O$	2124, 2132, 2138, 2152 2163	[S2]
$\{[Dy^{III}(H_2O)_2(4\text{-pyridone})_4][Co^{III}(CN)_6]\}\cdot 2H_2O$	2123, 2130, 2134, 2144, 2160	[S3]
$\{[Dy^{III}(H_2O)_2(4\text{-pyridone})_4][Rh^{III}(CN)_6]\}\cdot 2H_2O$	2133, 2138, 2144, 2156, 2176	[S3]
$\{[Ho^{III}(H_2O)_2(4\text{-pyridone})_4][Co^{III}(CN)_6]\}\cdot 2H_2O$	2123, 2130, 2134, 2144, 2160	[S4]
$\{[Ho^{III}(H_2O)_2(4\text{-pyridone})_4][Rh^{III}(CN)_6]\}\cdot 3H_2O$	2133, 2138, 2146, 2156, 2176	[S4]
$\{[Ho^{III}(H_2O)_2(4\text{-pyridone})_4][Ir^{III}(CN)_6]\}\cdot 2H_2O$	2131, 2136, 2145, 2156, 2180	[S4]

[S1] Chorazy, S.; Rams, M.; Nakabayashi, K.; Sieklucka, B.; Ohkoshi, S. White Light Emissive Dy^{III} Single-Molecule Magnets Sensitized by Diamagnetic $[Co^{III}(CN)_6]^{3-}$ Linkers. *Chem. Eur. J.* **2016**, *22*, 7371–7375.

[S2] Chorazy, S.; Zakrzewski, J. J.; Reczyński, M.; Nakabayashi, K.; Ohkoshi, S.; Sieklucka, B. Humidity driven molecular switch based on photoluminescent Dy^{III}Co^{III} single-molecule magnets. *J. Mater. Chem. C* **2019**, *7*, 4164–4172.

[S3] Wang, J.; Chorazy, S.; Nakabayashi, K.; Sieklucka, B.; Ohkoshi, S. Achieving white light emission and increased magnetic anisotropy by transition metal substitution in functional materials based on dinuclear Dy^{III}(4-pyridone)$[M^{III}(CN)_6]^{3-}$ (M = Co, Rh) molecules. *J. Mater. Chem. C* **2018**, *6*, 473–481.

[S4] Wang, J.; Zakrzewski, J. J.; Zychowicz, M.; Vieru, V.; Chibotaru, L. F.; Nakabayashi, K.; Chorazy, S.; Ohkoshi, S. Holmium(III) molecular nanomagnets for optical thermometry exploring the luminescence re-absorption effect. *Chem. Sci.* **2021**, *12*, 730–741.

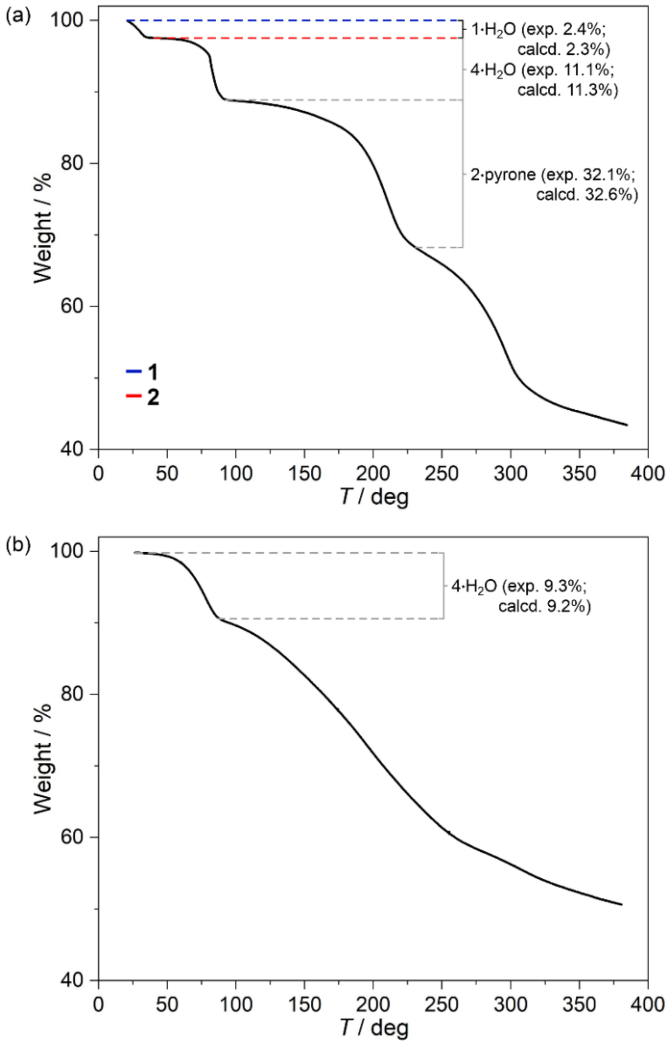

Figure S2. Thermogravimetric curves of **1** (*a*) and **2** (*b*) gathered in the temperature range of 25–375 °C. The experiments were conducted under a N_2 atmosphere with the heating rate of 1 °C·min^{-1}.

Comment to Figure S2

The dehydration process of **1** and the thermal stability of the resulting phase **2** was studied using a thermogravimetric (TG) method (Figure S2). The sample of **1** readily transforms to **2** under the N_2 flow even at room temperature. To slow down this process, the sample of **1** was measured using an Al_2O_3 pan equipped with a lid. Such a semi-closed crucible ensures that the dehydration process does not occur before the start of the TG measurement. It also leaves temperature as the most important dehydration-inducing factor while the direct influence of a nitrogen flow is limited due to the partial protection of the sample. This setup is also the closest possible to the conditions used a single crystal protected by Apiezon® N grease during the *in situ* dehydration in the diffractometer by using a liquid-nitrogen-cooled cryostat. Under such conditions, upon slow heating, the powder sample of **1** quickly loses its weight by 2.4% which perfectly corresponds to the removal of one crystallization water molecule per the {EuCo} formula unit (Figure S2a). As a result, the sample transforms into the crystalline phase **2** at the temperature of ca. 35 °C which remains stable upon further heating to ca. 60 °C under these experimental conditions. The observed short plateau is followed by an abrupt weight loss in the 70–90 °C range that can be correlated with the removal of both crystallization and coordinated water molecules (for water molecules per formula unit). Then, an anhydrous sample seems to exhibit overall weak stability as immediately a gradual decrease of the sample weight is detected. With an increasing temperature, the weight loss accelerates. Its further slowdown is observed presumably after the removal of two molecules of 2-pyrrolidone per formula unit at the temperature of ca. 230 °C. Above this point, the decomposition of a residual sample is observed.

The TG experiment was also performed for compound **2**. The most efficient method to carry out this measurement consists of inserting the sample of **1** into the nitrogen flow in a standard Al pan. It results in the fast structural transformation to phase **2** occurring almost immediately. Then, the TG experiment for the *in situ* prepared phase **2** was performed. The TG curve for **2** measured in such conditions exhibits the modified course, which is characterized by the lower stability of **2** and the broadening of the decomposition steps (Figure S2b). Here, the removal of all remaining four water molecules occurs at 50 °C. The anhydrous phase is generated at ca. 90 °C. From this point, an almost monotonous, gradual decrease of the sample mass is observed, which is presumably connected with the reactivity of the residual sample. At the highest temperatures, the different final products are obtained, when compared to the decomposition occurring in a semi-closed crucible. This can be ascribed to the presence of the large nitrogen excess in the second applied measurement setup.

Table S2. Selected structural parameters of Eu^{III} complexes in **1** and **2**.

Parameter	1	2
Eu1–O1	2.433(2) Å	2.500(3) Å
Eu1–O2	2.431(2) Å	2.421(3) Å
Eu1–O3	2.412(2) Å	2.398(3) Å
Eu1–O4	2.355(2) Å	2.376(3) Å
Eu1–O5	2.395(2) Å	2.406(3) Å
Eu1–O6	2.419(2) Å	2.407(3) Å
Eu1–O7	2.357(2) Å	2.358(3) Å
Eu1–N1	2.489(2) Å	2.484(4) Å
O1–Eu1–O2	76.58(5)°	71.8(1)°
O1–Eu1–O3	123.73(6)°	119.7(1)°
O1–Eu1–O4	69.51(5)°	67.1(1)°
O1–Eu1–O5	80.98(5)°	84.9(1)°
O1–Eu1–O6	137.21(5)°	139.5(1)°
O1–Eu1–O7	68.07(5)°	68.1(1)°
O1–Eu1–N1	141.64(6)°	141.7(1)°
O2–Eu1–O3	67.85(6)°	67.8(1)°
O2–Eu1–O4	73.18(6)°	72.3(1)°
O2–Eu1–O5	148.69(5)°	146.9(1)°
O2–Eu1–O6	136.64(5)°	136.7(1)°
O2–Eu1–O7	104.36(6)°	106.3(1)°
O2–Eu1–N1	94.53(6)°	95.5(1)°
O3–Eu1–O4	132.45(6)°	133.1(1)°
O3–Eu1–O5	143.45(6)°	145.3(1)°
O3–Eu1–O6	69.68(6)°	69.6(1)°
O3–Eu1–O7	80.05(6)°	82.6(1)°
O3–Eu1–N1	84.63(6)°	85.5(1)°
O4–Eu1–O5	78.68(6)°	77.1(1)°
O4–Eu1–O6	135.49(5)°	138.6(1)°
O4–Eu1–O7	136.76(6)°	132.9(1)°
O4–Eu1–N1	72.19(6)°	74.6 (1)°
O5–Eu1–O6	74.11(5)°	75.89(1)°
O5–Eu1–O7	86.85(6)°	85.3(1)°
O5–Eu1–N1	89.63(6)°	88.4(1)°
O6–Eu1–O7	76.23(5)°	75.0(1)°
O6–Eu1–N1	73.08(6)°	73.8(1)°
O7–Eu1–N1	148.88(6)°	148.8(1)°
Eu1–N1–C1	158.8(2)°	161.8(4)°
shortest Eu1↔Co1 distance	5.4261(4) Å	5.4583(9) Å
shortest Eu1↔Eu1 distance	9.0135(6) Å	8.112(1) Å

Table S3. Selected structural parameters of Co^{III} complexes in **1** and **2**.

Parameter	1	2
Co1–C1	1.893(2) Å	1.901(5) Å
Co1–C2	1.903(2) Å	1.905(5) Å
Co1–C3	1.898(2) Å	1.902(5) Å
Co1–C4	1.894(2) Å	1.895(5) Å
Co1–C5	1.895(2) Å	1.895(5) Å
Co1–C6	1.902(2) Å	1.895(5) Å
C1–N1	1.152(3) Å	1.154(6) Å
C2–N2	1.154(3) Å	1.151(6) Å
C3–N3	1.153(3) Å	1.150(6) Å
C4–C4	1.151(3) Å	1.148(6) Å
C5–N5	1.148(3) Å	1.152(6) Å
C6–N6	1.154(3) Å	1.146(6) Å
C1–Co1–C2	178.44(9)°	178.5(2)°
C3–Co1–C5	175.75(9)°	176.5(2)°
C4–Co1–C6	177.51(9)°	176.7(2)°
C1–Co1–C3	90.65(9)°	93.6(2)°
C1–Co1–C4	89.96(9)°	89.1(2)°
C1–Co1–C5	90.05(9)°	89.5(2)°
C1–Co1–C6	87.78(9)°	87.6(2)°
C2–Co1–C3	88.80(9)°	87.8(2)°
C2–Co1–C4	91.48(9)°	91.3(2)°
C2–Co1–C5	90.60(9)°	89.01(2)°
C2–Co1–C6	90.80(9)°	92.0(2)°
C3–Co1–C4	87.52(9)°	88.4(2)°
C3–Co1–C6	93.53(9)°	91.3(2)°
C4–Co1–C5	88.28(9)°	90.1(2)°
C5–Co1–C6	90.69(9)°	90.4(2)°

Table S4. Results of Continuous Shape Measure (CSM) analysis for $[Eu^{III}(H_2O)_3(pyrone)_4(NC)]^{2+}$ complexes in the crystal structures of **1** and **2**.

**Compound**	CSM parameters*			Geometry
	SAPR–8	TDD–8	BTPR–8	
**1**	2.839	1.109	1.620	TDD-8
**2**	3.052	1.566	1.579	TDD-8

* CSM parameters:
CSM SAPR–8 – the parameter related to the square antiprism geometry (D_{4d} symmetry)
CSM TDD–8 – the parameter related to the triangular dodecahedron geometry (D_{2d} symmetry)
CSM BTPR–8 – the parameter related to the biaugmented trigonal prism geometry (C_{2v} symmetry)
CSM = 0 for the ideal geometry and the increase of CSM parameter corresponds to the increasing distortion from the ideal polyhedron.

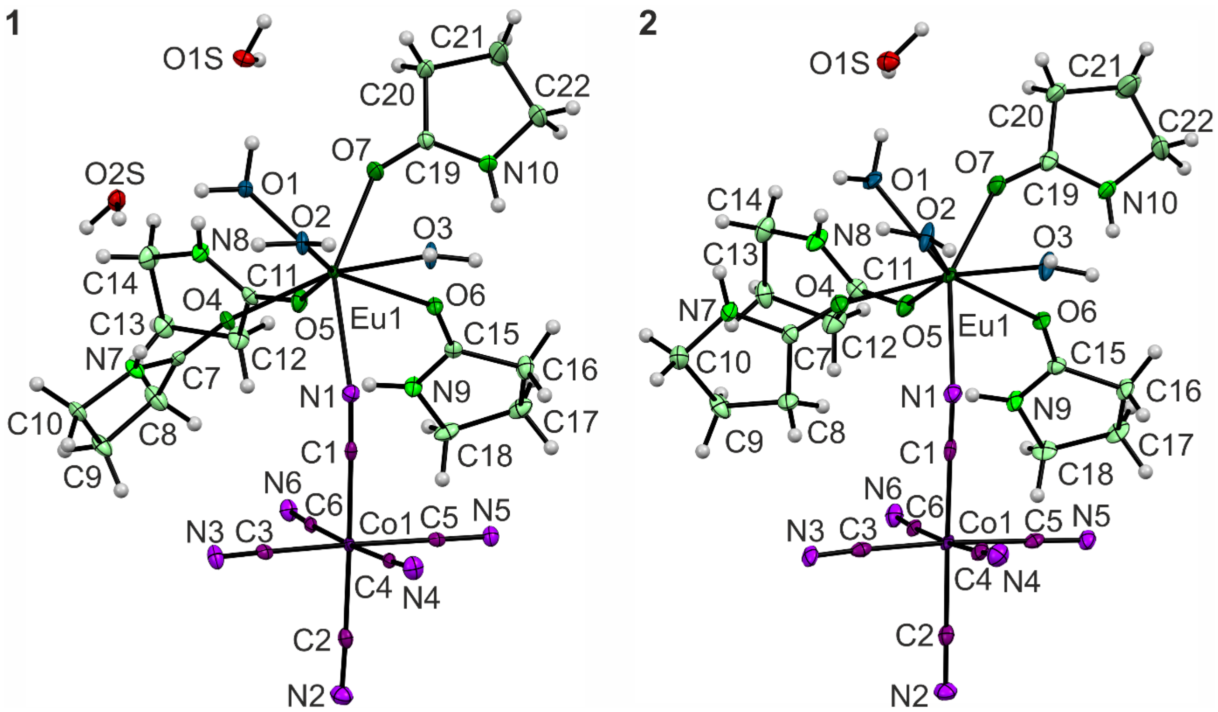

Figure S3. Comparison between the asymmetric units of **1** (left) and **2** (right). All non-hydrogen atoms were labelled in the figure while hydrogen atoms were shown as light grey spheres. Thermal ellipsoids are presented at the 50% probability level while hydrogen atoms are drawn as fixed-sized spheres with a 0.15 Å radius.

Table S5. Detailed parameters of the hydrogen bonds in the crystal structure of **1**.

**H bond**	**D–A distance [Å]**	**H–A distance [Å]**	**D–H→A angle [°]**
O1–H1→O1S	2.723(2)	1.79(2)	166(3)
O1–H2→O2S	2.717(2)	1.77(1)	171(3)
O2–H3→N3	2.813(2)	1.89(1)	173(3)
O2–H4→N6	2.862(2)	1.96(2)	162(3)
O3–H5→N5	2.761(2)	1.83(1)	176(3)
O3–H6→N6	2.878(3)	1.96(2)	167(3)
N7–H7N→N3	3.193(3)	2.43	145.5
N8–H8N→O1	3.009(2)	2.25	144.9
N8–H8N→O2S	3.065(3)	2.49	123.8
N9–H9N→O5	2.883(3)	2.21	132.6
N10–H10N→N5	3.151(3)	2.43	139.3
N10–H10N→O6	3.029(3)	2.37	131.5
O1S–H1SA→N2	2.857(3)	1.95(1)	174(3)
O1S–H1SB→N4	2.778(3)	1.89(2)	164(3)
O2S–H2SA→N3	3.188(3)	2.35(2)	155(3)
O2S–H2SB→O1S	2.874(2)	2.01(2)	160(3)

Table S6. Detailed parameters of the hydrogen bonds in the crystal structure of **2**.

**H bond**	**D–A distance [Å]**	**H–A distance [Å]**	**D–H→A angle [°]**
O1–H1→O1S	2.628(5)	1.64(2)	174(8)
O1–H2→N3	2.973(5)	2.02(3)	160(6)
O2–H3→N3	2.788(5)	1.91(5)	146(7)
O2–H4→N6	2.828(5)	1.95(5)	147(7)
O3–H5→N5	2.741(5)	1.76(2)	178(7)
O3–H6→N6	2.869(5)	1.92(3)	161(6)
N8–H8N→O1	2.925(5)	2.11	153.5
N9–H9N→O5	3.008(5)	2.33	134.4
N10–H10N→N5	3.155(6)	2.46	136.6
N10–H10N→O6	3.004(5)	2.34	132.3
O1S–H1SA→N2	2.837(5)	1.90(2)	165(7)
O1S–H1SB→N4	2.803(5)	1.85(2)	176(8)

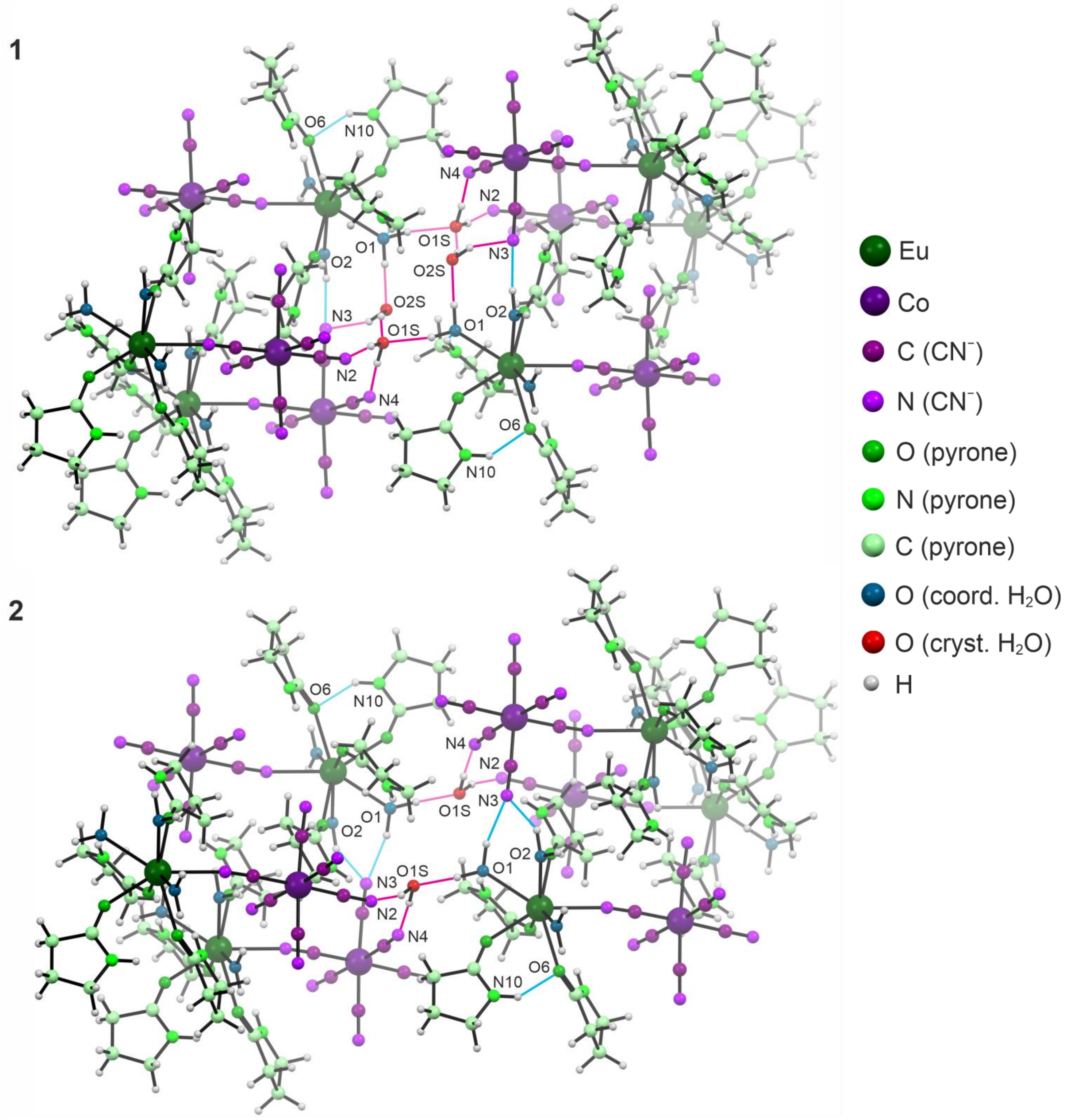

Figure S4. Detailed views of hydrogen bonds detected within the supramolecular networks of **1** (upper panel) and **2** (bottom panel). The hydrogen bonds related to the crystallization water molecules were presented as pink lines while the other visible hydrogen bonds were drawn using light blue lines. The color code for all atoms is presented on the right side. The related parameters of the H-bonding contacts are gathered in Table S5 (**1**) and Table S6 (**2**).

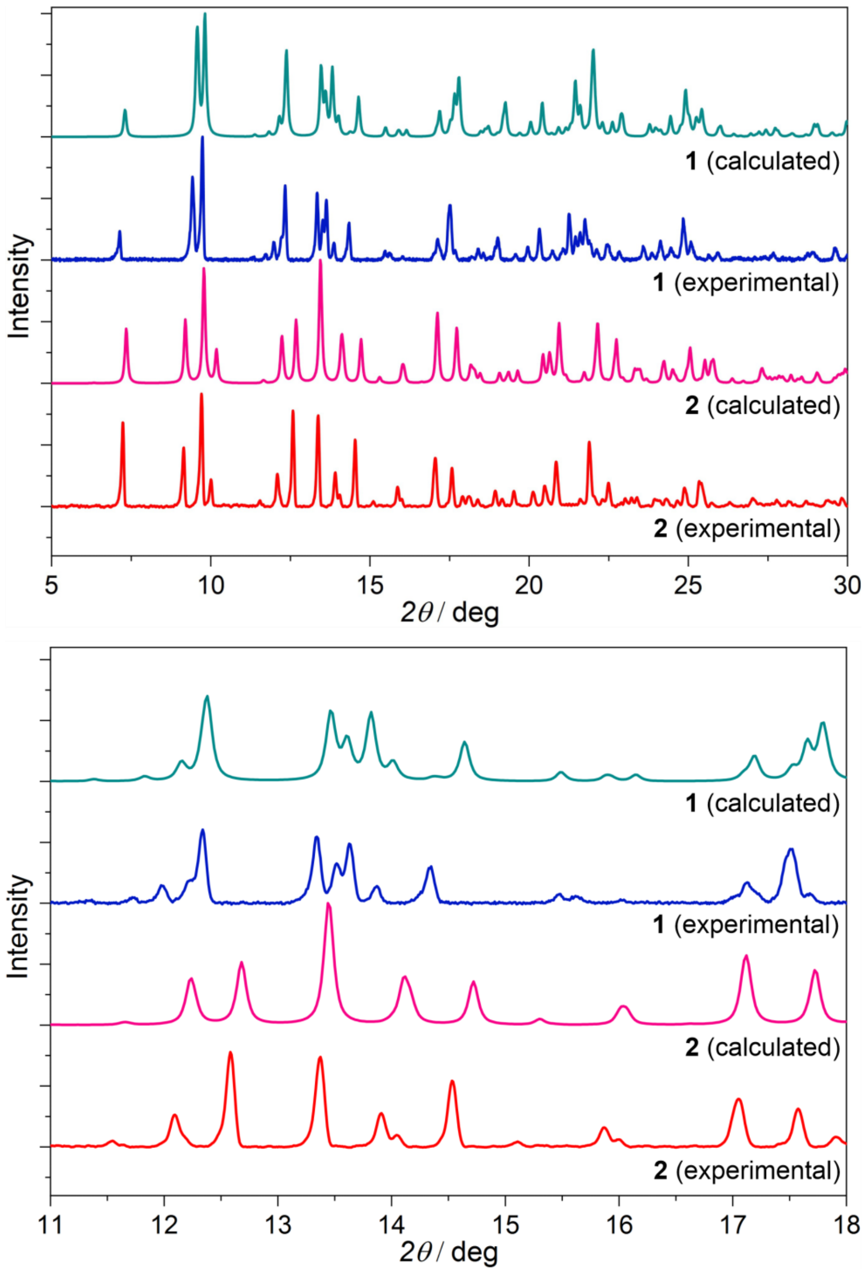

Figure S5. Powder X-ray diffraction (P-XRD) patterns for **1** and **2** measured at room temperature (top) together with the enlargement of the representative range of 11–18° of 2θ angle (bottom). The respective P-XRD patterns of **1** and **2**, calculated on the basis of the structural models achieved from the single-crystal X-ray (SC-XRD) structural analysis (T = 100 K), were also presented for comparison. Note that there are some differences (shifts of the peaks, modified splitting of the peaks) between experimental and calculated P-XRD patterns but this can be mainly ascribed to the temperature effect. In the experimental P-XRD pattern of **1**, there is no significant peak which cannot be assign to one of the peak from simulated pattern (see, for instance, the enlargement for the representative range of 11–18° of 2θ angle) indicating that the hydrated phase of **1** (prepared under the high humidity conditions, see Experimental section) does not contain significant impurity of the dehydrated 2. On the other hand, in the experimental P-XRD pattern of **2**, there are some peaks noticeably splitted when compared with the calculated pattern but they cannot be undoubtedly assign to any of the peaks of **2**. Nevertheless, a minor impurity of the hydrated phase **1** in the measured powder sample of **2** cannot be neglected which can be related to the imperfect dehydration of the sample in the glove-box chamber before the P-XRD experiment (see Experimental section for details regarding the preparation of the samples for P-XRD studies).

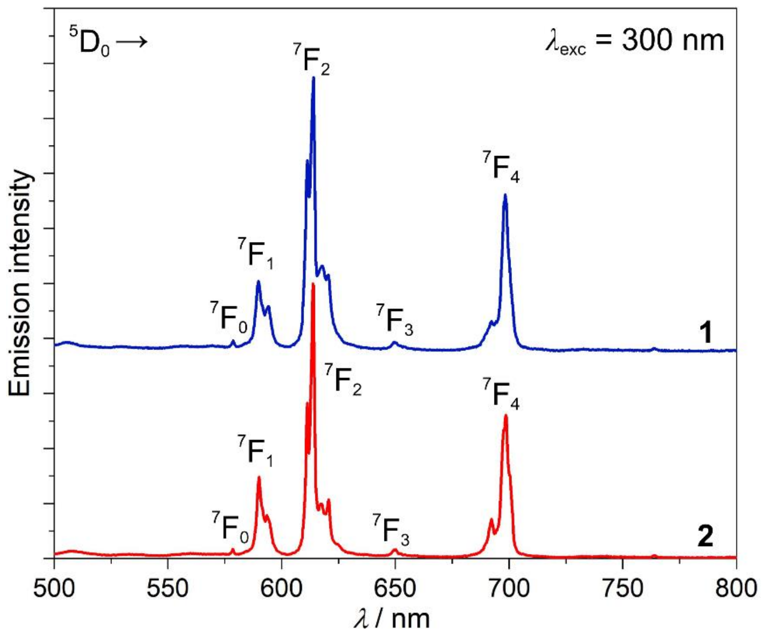

Figure S6. Solid-state emission spectra of **1** and **2** gathered at room temperature upon the 300 nm irradiation.

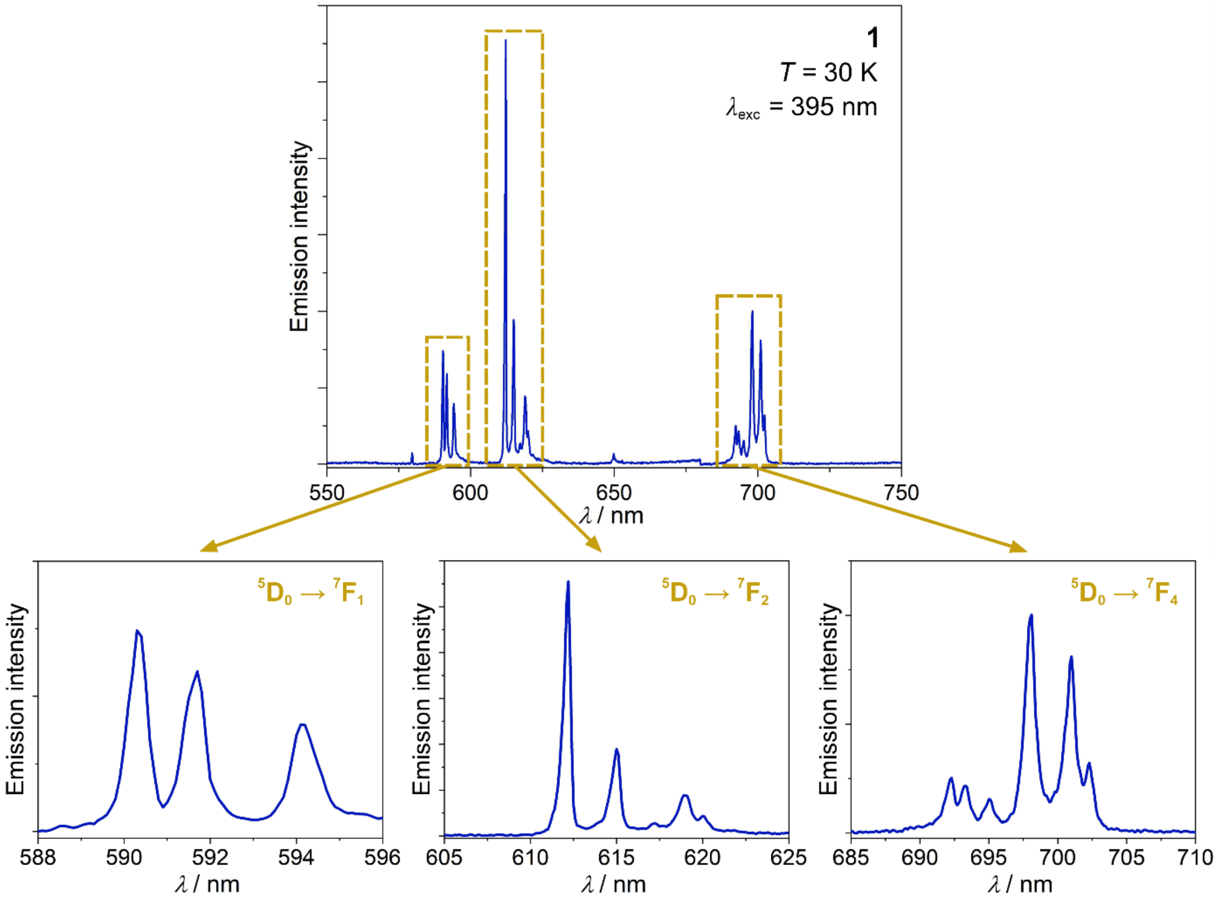

Figure S7. Solid-state emission spectrum of **1** gathered at 30 K upon the 395 nm irradiation with the enlargement of three main emissive bands shown with the labelling of the respective f-f electronic transitions.

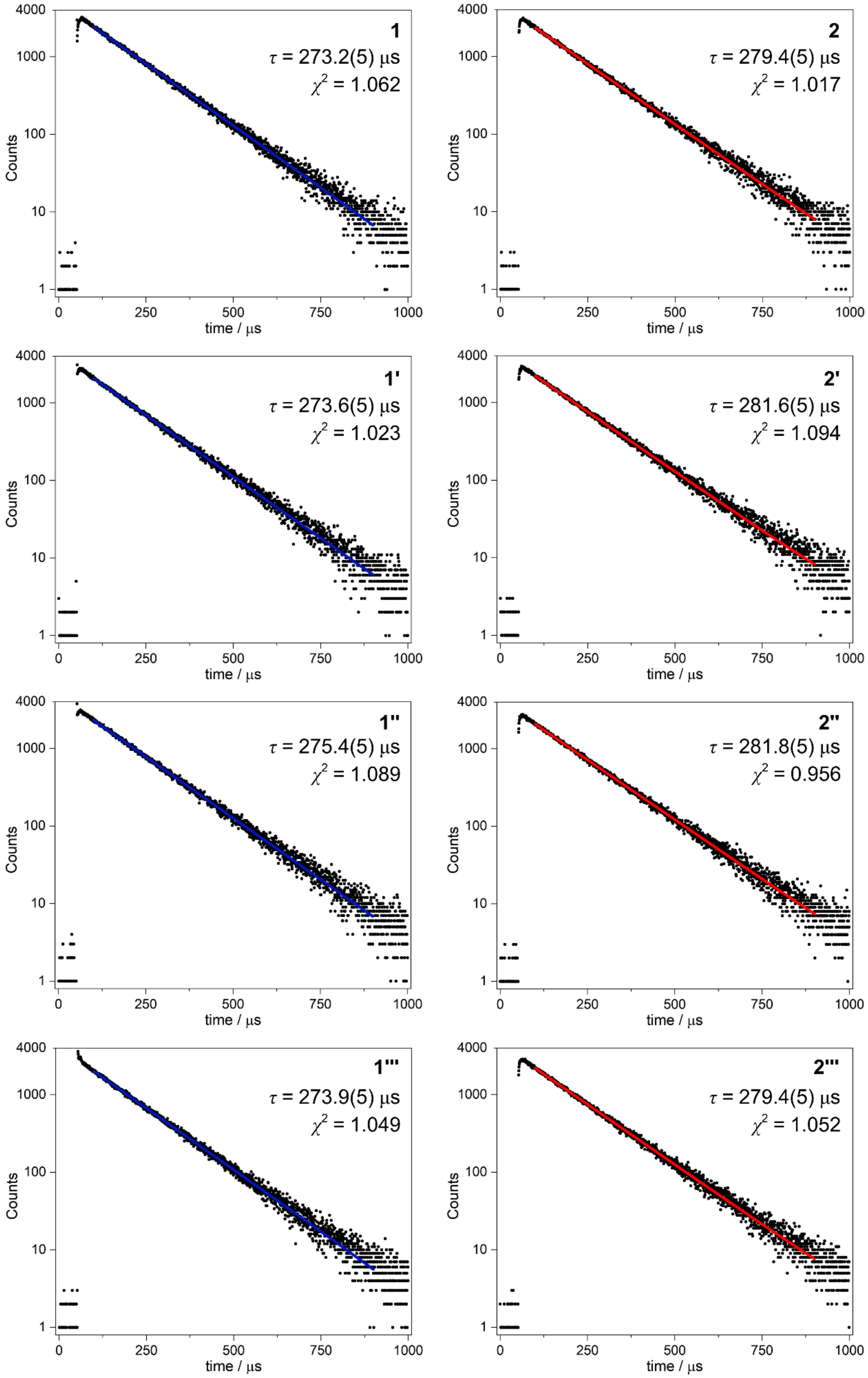

Figure S8. Emission decay profiles of **1** (left panel) and **2** (right panel) in the subsequent cycles of hydration and dehydration. Presented decay profiles were measured using λ_{exc}= 395 nm and λ_{em}= 611 nm. The black points are the experimental data while the colored solid lines show the best-fit curve using a mono-exponential decay function. The emission lifetimes are presented on the graphs together with the fitting coefficient (χ^2).
